# Assessing risk for HIV infection among adolescent girls in South Africa: an evaluation of the VOICE risk score (HPTN 068)

**DOI:** 10.1002/jia2.25359

**Published:** 2019-07-28

**Authors:** Danielle Giovenco, Audrey Pettifor, Catherine MacPhail, Kathleen Kahn, Ryan Wagner, Estelle Piwowar‐Manning, Jing Wang, James P Hughes

**Affiliations:** ^1^ Department of Epidemiology University of North Carolina at Chapel Hill Chapel Hill NC USA; ^2^ School of Health and Society University of Wollongong Wollongong New South Wales Australia; ^3^ MRC/Wits Rural Public Health and Health Transitions Research Unit (Agincourt) Acornhoek South Africa; ^4^ Department of Pathology Johns Hopkins University Baltimore MD USA; ^5^ Fred Hutchinson Cancer Research Center Seattle WA USA; ^6^ Department of Biostatistics University of Washington Seattle WA USA

**Keywords:** HIV, AIDS, adolescent, women, risk assessment, pre‐exposure prophylaxis

## Abstract

**Introduction:**

To maximize impact and minimize costs, antiretroviral pre‐exposure prophylaxis (PrEP) interventions should be offered to those at highest risk for HIV infection. The risk score derived from the VOICE trial is one tool currently being utilized to determine eligibility in adolescent PrEP trials in sub‐Saharan Africa. This study is aimed at evaluating the utility of the risk score in predicting HIV incidence among a cohort of adolescent girls in rural South Africa.

**Methods:**

We utilized data from HIV Prevention Trials Network (HPTN) 068, a phase III randomized controlled trial conducted in rural Mpumalanga province, South Africa. School‐attending young women aged 13 to 20 years were enrolled into the trial from 2011 to 2012 and followed for up to three years. A risk score based on individual‐level risk factors measured at enrolment was calculated for HPTN 068 participants who completed a one‐year follow‐up visit and were HIV seronegative at enrolment. Possible scores ranged from 0 to 10. A proportional hazards model was then used to determine if risk score at enrolment was predictive of incident HIV infection at follow‐up and an area under the curve analysis was used to examine the predictive ability of the score.

**Results and Discussion:**

The risk score had limited variability in the HPTN 068 sample. Scores ≥5 identified 85% of incident infections from 94% of the sample, compared to the VOICE sample in which scores ≥5 identified 91% of incident infections from only 64% of participants. The risk score did not predict HIV incidence after one year of follow‐up (hazard ratio = 1.029; 95% confidence interval (CI): 0.704, 1.503, *p* = .884) and showed poor predictive ability (area under the curve = 0.55; 95% CI: 0.44, 0.65). Certain individual risk factors that comprise the risk score may be context specific or not relevant for adolescent populations. Additional factors should be considered when assessing risk for the purposes of determining PrEP eligibility.

**Conclusions:**

The VOICE risk score demonstrated low utility to predict HIV incidence in the HPTN 068 sample. Findings highlight the need for an age and developmentally appropriate tool for assessing risk for HIV infection among adolescents. Use of the VOICE risk score for determining PrEP eligibility in younger populations should be carefully considered.

## 1 Introduction

Biomedical HIV prevention technologies, such as antiretroviral pre‐exposure prophylaxis (PrEP), hold enormous potential to substantially reduce HIV acquisition in high‐risk populations globally. To maximize impact and minimize costs, PrEP interventions should be offered to those at highest risk for HIV infection. HIV risk assessment tools have been generated for several key populations, including heterosexual HIV serodiscordant couples 1, men who have sex with men 2, 3, and pregnant and postpartum women 4.

In attempts to identify African women at greatest risk for HIV infection, Balkus *et al*. 5 developed and validated a risk assessment tool to predict one‐year risk of HIV acquisition among African women in generalized HIV epidemic settings, where PrEP scale‐up is especially warranted. The authors analysed data from three randomized placebo‐controlled trials of biomedical HIV prevention technologies, including the VOICE trial, HIV Prevention Trials Network (HPTN) 035, and FEM‐PrEP. The VOICE trial assessed the safety and effectiveness of daily oral tenofovir disoproxil fumarate (TDF), oral TDF/emtricitabine (FTC), and 1% vaginal tenofovir gel as PrEP for HIV prevention 6. HPTN 035 and FEM‐PrEP tested the effectiveness of a microbicide gel and oral TDF/FTC respectively 7, 8. Baseline demographic, behavioural, clinical and self‐reported male partner characteristics from the VOICE study were used to identify a discrete set of easy‐to‐measure characteristics that were thought to be predictive of HIV acquisition. These individual‐level risk factors were assigned a value and a total risk score for each participant was calculated by taking the sum of values for each risk factor, with possible scores ranging from 0 to 11. The risk score was then externally validated by separately applying it to the HPTN 035 and FEM‐PrEP study populations. The VOICE trial, HPTN 035, and FEM‐PrEP all included women ≥18 years of age (Median ages = 23‐25 years).

Adolescents and young adults aged 15‐24 years in sub‐Saharan Africa account for over one‐third all new infections, with over twice as many new infections among young women than young men 9. Adolescents <18 years of age, however, remain inadequately represented in biomedical HIV prevention research posing a significant challenge for integrating strategies such as PrEP into effective combination prevention packages 10. To address this need, several open‐label PrEP demonstration studies aimed at understanding key barriers and facilitators to PrEP acceptability, uptake, and adherence among adolescents at highest risk for HIV infection are currently planned or ongoing in sub‐Saharan Africa 11. The risk score derived from the VOICE trial is one tool currently being utilized to determine PrEP eligibility in adolescent trials 12. Therefore, this research was aimed at validating the risk score for use in a population of adolescent girls in rural South Africa.

## 2 Methods

Data from HPTN 068 was utilized for this analysis. HPTN 068 was a phase III randomized controlled trial conducted in Mpumalanga province, South Africa within the Agincourt Health and socio‐Demographic Surveillance System site. This region is a rural area characterized by high HIV prevalence, poverty and migration for work 13. The aim of the study was to assess the impact of a cash transfer, conditioned on school attendance, on HIV incidence. School‐attending young women aged 13 to 20 years, who were not married or pregnant, were enrolled into the study between March 2011 and December 2012 and followed for up to three years. Written informed consent from a primary caregiver (parent or legal guardian) and written informed consent/assent from each young woman was obtained prior to participation. More information on HPTN 068 study methods are detailed elsewhere 14, 15.

The risk score derived from the VOICE trial, which incorporates age, living with a primary partner, having a partner provide financial or material support, having partner who has other partners, any alcohol use in the past three months, and herpes simplex virus type 2 (HSV‐2) serostatus, was calculated at baseline for participants with complete data for individual‐level risk factors who were HIV seronegative. HPTN 068 participants were asked to provide information on their current partner(s). Participants were asked if they were currently living with each partner (yes/no), if they were receiving financial (“has [your partner] ever given you money?”) or material (“has [your partner] ever given things, like groceries, clothes or airtime, that help you get by”) support from each partner (yes/no), and if each partner had other sexual partners (yes/no/don't know). If HPTN 068 participants reported “yes” or “don't know” for least one partner, they were categorized as such for each risk factor. HPTN 068 did not collect data on curable sexually transmitted infections (STIs); therefore, the full risk score was adapted to exclude this risk factor (range = 0‐10). See Appendix for the full risk score derived from the VOICE trial.

Consistent with the VOICE risk score derivation and validation analyses, and when we hypothesized that the predictive ability of baseline risk factors would be strongest, we examined the relationship between baseline risk score and HIV incidence after one year of follow‐up. One‐year HIV incidence was observed for participants with a follow‐up visit occurring between 6 and 18 months post‐baseline. The number of incident infections was examined by risk score and a proportional hazards model was used to determine if baseline risk score was predictive of incident HIV infection. The area under the receiver operating characteristic curve (AUC) was used to assess the performance of the risk score in the HPTN 068 study population and the sensitivity and specificity at different risk score cut‐points were calculated.

## 3 Results and discussion

In the HPTN 068 sample, 99% of participants had complete baseline data for individual‐level risk factors (N = 2503/2533). Included in the analysis were 2178 participants with a one‐year follow‐up visit who were HIV seronegative at baseline. Participant demographic and behavioural characteristics are shown in Table 1. The median age of participants was 15 years (interquartile range = 14‐17 years). The majority of participants (89%) were under 18 years of age. Approximately 31% of participants had a “boyfriend or main partner,” 24% had at least one sex partner in their lifetime, and 22% had at least one sex partner in the past three months.

**Table 1:** Participant demographic and behavioural characteristics

	Total (N = 2178)^a^
Age	15 (14‐17)
School grade	
Grade 8 (13‐15 years)	567 (26.03)
Grade 9 (14‐16 years)	606 (27.82)
Grade 10 (16‐17 years)	600 (27.55)
Grade 11 (17‐18 years)	405 (18.6)
Prior pregnancy^b^	169 (7.76)
Current boyfriend or main partner^c^	680 (31.22)
Lifetime sexual partners	
0	1636 (75.60)
1	284 (13.12)
≥2	244 (11.28)
Missing (refused to answer)	14
Sexually active (past three months)	467 (21.57)
Missing (refused to answer)	13

^a^Data are median (interquartile range) or N (%); ^b^participants enrolled in HPTN 068 could not be currently pregnant; ^c^includes non‐sexual partners.

The distribution of HPTN 068 participants across individual risk factors that comprise the risk score at baseline are presented in Table 2. All participants were under 25 years of age. Approximately 3% reported that they were living with a primary partner, 21% were receiving financial or material support from a partner, and 19% reported having a partner with other partners. Furthermore, 9% of the sample had used alcohol in the past three months and 4% were HSV‐2 seropositive. Univariate analyses of individual risk factors and HIV incidence revealed that having a partner provide financial or material support was associated with increased risk for HIV infection (*p* < .001). This finding is dissimilar to the VOICE trial, where having a partner provide financial or material support was found to be protective against HIV infection.

**Table 2:** Key baseline risk factors in the HPTN 068 study population

Individual risk factors	Risk score	HPTN 068 (N = 2178)	Univariate analysis
		N (%)	HR (95% CI)
Age			
<25 years	2	2178 (100.00)	–
≥25 years	0	0 (0.00)	1.00
Living with primary partner			
No	2	2103 (96.56)	0.43 (0.13, 1.41)
Yes	0	75 (3.44)	1.00
Partner provides financial/material support			
No	1	1721 (79.02)	0.31 (0.16, 0.62)^a^
Yes	0	457 (20.98)	1.00
Primary partner has other partners			
Yes/don't know	2	407 (18.69)	1.69 (0.82, 3.50)
No	0	1771 (81.31)	1.00
Alcohol use in past three months			
Yes	1	192 (8.82)	1.36 (0.48, 3.88)
No	0	1986 (91.18)	1.00
HSV‐2 seropositive			
Yes	2	77 (3.54)	2.82 (0.98, 8.10)
No	0	2101 (96.46)	1.00

HR, hazard ratio; CI, confidence interval.

a *p* < .05.

**Figure 1:**
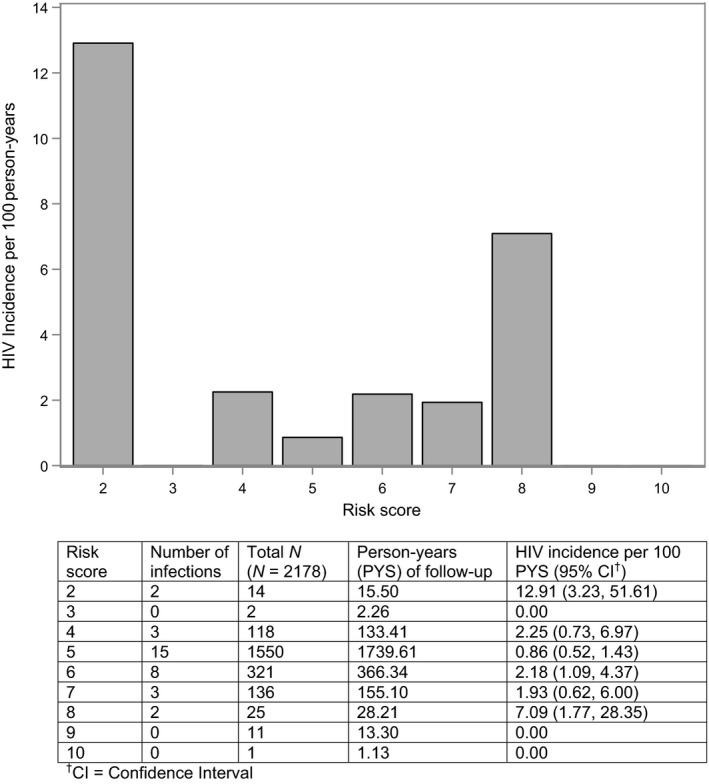
One‐year HIV incidence by risk score CI's calculated using the quadratic approximation to the Poisson log likelihood for the log‐rate parameter.

There were 33 HIV seroconversions during 2455 person‐years of follow‐up (1.34% incidence over an average follow‐up period of 1.13 years). HIV incidence by risk score is shown in Figure 1. A majority (71%) of participants received a risk score of 5 (range = 2‐10). Scores ≥5 identified 85% of incident infections from 94% of the sample. In the VOICE sample, scores ≥5 identified 91% of incident infections from only 64% of participants. Therefore, a larger proportion of participants were categorized as being “at risk” (with a risk score cut‐point of ≥5) and, among these participants, fewer incident infections were identified in the HPTN 068 cohort compared with the VOICE cohort. To maximize impact and minimize costs, biomedical HIV prevention technologies should be offered to those at highest risk for HIV infection. With a cut‐point of ≥5, for example, 94% of the HPTN 068 sample would be eligible for PrEP.

Risk score did not predict HIV incidence (hazard ratio (HR) = 1.029, 95% confidence interval (CI): 0.704, 1.503, *p* = 0.884). Furthermore, among a subset of 528 adolescents included in the analysis who had at least one lifetime sexual partner at baseline, there were 18 HIV seroconversions during 603 person‐years of follow‐up (2.99% incidence). The risk score still did not predict HIV incidence (HR = 0.877, 95% CI: 0.635, 1.211, *p* = 0.424).

Among the 2,178 participants included in the analysis, the AUC for the risk score was 0.55 (95% CI: 0.44, 0.65). In the derivation and initial validation cohorts 5, all of which included women 18 years of age or older, the risk score performed more accurately. The AUC for the risk score in the HPTN 068 sample was lower than the AUC observed in the VOICE study (AUC = 0.71, 95% CI: 0.68, 0.74) and an external validation in HPTN 035 (AUC = 0.70, 95% CI: 0.65, 0.75), and similar to an external validation in FEM‐PrEP (AUC = 0.58, 95% CI: 0.51, 0.65). The predictive ability of the risk score in the HPTN 068 cohort was also lower than a single‐item question identifying sexually active adolescent girls (AUC = 0.65, 95% CI: 0.57, 0.74).

An analysis of additional performance characteristics for the full sample revealed that adequate sensitivity was only maintained under very low specificity. A risk score cut‐point of ≥5 had a sensitivity and specificity of 85% and 6% respectively (see Table 3), with 94% PrEP eligibility. With a cut‐point of >5, however, only 23% of the sample would be eligible for PrEP, with a sensitivity of 39% and specificity of 78%. Given the large proportion of HPTN 068 participants who received a risk score of exactly 5 (71%), there is no clear cut‐point for identifying adolescents at risk for HIV infection using the VOICE risk score. In the VOICE derivation cohort, a risk score cut‐point of ≥5 had a sensitivity of 91% and a specificity of 38%. Furthermore, the HPTN 035 external validation cohort, which did not include alcohol use in their risk score (maximum score = 10), had a sensitivity and specificity of 58% and 71% respectively, with a cut‐point of ≥5. Lastly, the FEM‐PrEP validation cohort, which excluded risk factors such as alcohol use, having a partner provide financial or material support, and HSV‐2 serostatus (maximum score = 4), had a sensitivity and specificity of 83% and 31% respectively, with a cut‐point of ≥2. While the desired sensitivity and specificity would be highly dependent on the resource constraints and the availability of PrEP in the setting in which it is being prescribed, there would ideally be a less significant tradeoff.

**Table 3:** Detailed sensitivity and specificity by risk score cut‐point

Cut‐point	Incident infections (n = 33)		Non‐infections (n = 2145)		Sensitivity, %	Specificity, %
	True positives	False negatives	False positives	True negatives		
≥2	33	0	2145	0	100.00	0.00
≥3	31	2	2133	12	93.94	0.56
≥4	31	2	2131	14	93.94	0.65
≥5	28	5	2016	129	84.85	6.01
≥6	13	20	481	1664	39.39	77.58
≥7	5	28	168	1977	15.15	92.17
≥8	2	31	35	2110	6.06	98.37
≥9	0	33	12	2133	0.00	99.44
≥10	0	33	1	2144	0.00	99.95
>10	0	33	0	2145	0.00	100.00

Findings show that certain individual‐level risk factors from the VOICE risk score may not be relevant for adolescent populations. For example, the entire HPTN 068 study population was under 25 years of age (median age at baseline = 15 years). Furthermore, the individual risk factors derived from the VOICE study may not be relevant to the adolescent developmental trajectory. No HPTN 068 participants were married (per inclusion criteria), less than one‐third had a primary partner, and less than one‐quarter were sexually active. Therefore, questions related to cohabitation, partner support, and partner behaviour may not be appropriate. Even when only sexually active adolescents were observed, however, the risk score still did not predict HIV incidence.

The VOICE risk‐score may also not be relevant for the HPTN 068 context. HPTN 068 enrolled adolescent girls from a rural region in Mpumalanga province, an area characterized by high HIV prevalence, poverty and migration for work. Adolescent girls and young women in this context may navigate partnerships differently than South African populations more generally. While HPTN 068, the VOICE study, and the validation cohorts all included women from South Africa, there may be variability in risk factors between study sites.

Additional risk factors should be considered when assessing risk for HIV infection in adolescent populations. For example, sexual debut, having multiple sexual partners (both primary and casual partner types), and inconsistent condom use should be explored as potential risk factors. Furthermore, risk assessment tools for adolescents should consider context specific factors in addition to age, gender, and developmentally appropriate factors. For example, research has demonstrated the protective effect of schooling on HIV risk (and risk associated with dropping out of school), especially among adolescent girls in rural contexts 16, 17, 18. Furthermore, research among adolescent girls and young women in this context has shown that having older partners 19, 20 and engaging transactional sex 21, 22 are predictive of HIV incidence. Findings from our univariate analysis also demonstrate that having a partner who provides financial or material support is associated with increased risk for HIV infection. In comparison to the VOICE trial, where this factor was protective of HIV risk and suggested to be a proxy for relationship stability, having a partner provide financial or material support may be an indicator of higher risk transactional sex among younger populations.

Lastly, in addition to identifying those at greatest risk for HIV infection, another challenge is identifying adolescents who perceive themselves to be at risk and thus will be likely to adhere to a biomedical prevention technology such as PrEP 23, 24. By balancing interest in PrEP based on perceptions of risk and risk for HIV infection itself, a cost‐effective approach to identifying adolescents who are most likely to benefit from the intervention can be implemented.

There are several potential limitations to this research. First, response bias is likely to occur for sensitive questions, especially among adolescent participants. For example, HPTN 068 participants may be more likely to under‐report sexual activity and substance use compared to older participants in the VOICE cohort. Second, this analysis utilized data from a trial conducted from 2011 to 2014. While the data were collected several years prior to these analyses, HPTN 068 offered a large, longitudinal cohort of adolescent girls in rural South Africa with minimal missing data and unmeasured confounding that we feel is still relevant to new and ongoing PrEP implementation efforts. However, given the use of a secondary data source, there are likely differences in how individual risk factors were defined. Furthermore, HPTN 068 did not collect data on curable STIs, a risk factor identified in the VOICE analysis. Lastly, HPTN 068 enrolled young women who were currently in school. Therefore, this population may be at lower risk for engaging in certain risk behaviours compared to young women who were not in school or who have had less schooling, resulting in possible selection bias.

## 4 Conclusions

We found that a risk score developed among adult women to predict HIV incidence and that is being used to target PrEP use in some settings was not helpful in identifying those at highest risk in a population of adolescent girls in South Africa. Findings highlight the need for an age and developmentally appropriate tool for assessing risk for HIV infection among adolescents in this context. Use of the VOICE risk score for determining PrEP eligibility in younger populations should be carefully considered.

## Competing interests

The authors declare that they have no competing interests.

## Authors’ contributions

AP, KK, CM, RW, JPH and EPM designed and conducted HPTN 068, the dataset utilized for this research. AP and DG designed the current study. DG, JW and JPH analysed the data. DG and AP wrote the manuscript. All authors have read, contributed to and approved the final manuscript.

## Appendix 1

### 1.1

**Table A1:** VOICE risk score

Baseline characteristics	Risk score
1. Age	
<25 years	2
≥25 years	0
2. Married or living with husband or primary partner	
No	2
Yes	0
3. Partner provides financial/material support	
No	1
Yes	0
4. Primary sex partner has other partners	
Yes	2
No	0
Don't know	2
5. Alcohol use in past three months	
Yes	1
No	0
6. HSV‐2 seropositive	
Yes	2
No	0
7. Any curable STI^a^	
Yes	1
No	0
Maximum VOICE risk score	11

a Chlamydia, gonorrhea, trichomoniasis, or syphilis.
